# A multi-center, randomized, double-blind, placebo-controlled trial to evaluate the efficacy and safety of Fuzheng Yangxin Granule in treating heart failure with preserved ejection fraction (Qi-Yin deficiency and blood stasis syndrome): study protocol

**DOI:** 10.3389/fcvm.2025.1514181

**Published:** 2025-04-29

**Authors:** Jingjing Chen, Zian Yan, Jiacong Wang, Lijun Guo, Zhonghui Jiang, Fangfang Wang, Ruina Bai, Xiaochang Ma

**Affiliations:** 1 School of Clinical Medicine, Xiyuan Hospital, Beijing University of Chinese Medicine, Beijing, China; 2 Cardiovascular Department, Xiyuan Hospital, China Academy of Chinese Medical Sciences, Beijing, China; 3 Cardiovascular Disease Center, Xiyuan Hospital, National Clinical Research Center for Chinese Medicine Cardiology, Beijing, China

**Keywords:** heart failure with preserved ejection fraction, Fuzheng Yangxin Granule, traditional Chinese medicine, clinical trial protocol, herbal medicine, randomized controlled trial

## Abstract

**Introduction:**

Heart failure with preserved ejection fraction (HFpEF) is a widespread public health issue worldwide. Despite recent advances in pharmacologic treatments and the introduction of new diagnostic approaches, HFpEF remains underdiagnosed and under-recognized in clinical practice. Traditional Chinese medicine (TCM) may offer a potentially effective treatment for HFpEF. Nevertheless, few clinical trials employ rigorous research methodologies to evaluate the efficacy and safety of TCM in treating HFpEF. Consequently, we propose to assess the hypothesis that patients with HFpEF may benefit from Fuzheng Yangxin Granule (FZYX) and evaluate its safety in a rigorously designed clinical trial.

**Methods:**

This multicenter, double-blind, randomized controlled trial will be conducted across seven tertiary hospitals in China. We will enroll 150 participants aged 18–80 years with confirmed HFpEF (Qi-Yin deficiency and blood stasis syndrome) meeting inclusion criteria. Participants will be randomly assigned (1:1) to the FZYX group or the placebo group, ​with both groups receiving standardized Western medical therapy according to the National Heart Failure Guideline 2023. The 12-week intervention phase will be followed by 40-week safety follow-up. The primary outcome will be maximal peak oxygen uptake (peak VO_2_). Secondary outcomes will include composite endpoint events, all-cause mortality, 6-minute walking distance (6MWD), New York Heart Association (NYHA) functional class, serum N-terminal pro-B-type natriuretic peptide (NT-proBNP), echocardiographic variables, Minnesota Living with Heart Failure Questionnaire (MLHFQ) score, TCM syndrome scores, and the FRAIL scale.

**Discussion:**

The objective of this study is to evaluate the efficacy and safety of FZYX in treating HFpEF (Qi-Yin deficiency and blood stasis syndrome), thereby providing a high-quality, reliable evidence-based foundation for clinical practice.

**Clinical Trial Registration:**

China Clinical Trial Registry (ChiCTR2400087293), Registered on July 24, 2024.

## 1 Introduction

Heart failure with preserved ejection fraction (HFpEF) is not merely a single pathological diagnosis but a complex clinical syndrome characterized by a left ventricular ejection fraction (LVEF) ≥ 50%, along with symptoms such as dyspnea and weakness (1). HFpEF is estimated to account for approximately 50% of all heart failure cases (2), with a 5-year survival rate of 35%–40% following the first hospitalization (3). And a 5-year mortality rate comparable to that of heart failure with reduced ejection fraction (HFrEF) (4).

Due to the heterogeneous pathophysiological mechanisms of HFpEF, evidence-based treatment and diagnosis rely primarily on clinical symptoms, signs, and evidence of structural and/or functional cardiac abnormalities. Despite significant efforts by scholars to develop treatments for HFpEF, translating preclinical experimental medicine into clinical practice remains a challenge. For example, trials targeting the renin-angiotensin system (RAS) [PEP-CHF trial (5), I-PRESERVE study (6), CHARM-Preserved study (7)], mineralocorticoid receptor antagonists (MRA) [TOPCAT study (8)], beta-blockers [OPTIMIZE-HF study (9)], and digitalis [DIG-PEF study (10)] have been conducted. None of these trials have demonstrated that conventional heart failure treatments effectively reduce mortality, readmission rates, or improve prognosis in HFpEF. These include trials investigating nitrate drugs [NEAT-HFPEF study (11)], inhaled nitrites [INDIE-HFpEF study (12)], soluble guanylate cyclase agonists [DILATE-1 study (13)], phosphodiesterase-5 inhibitors [RELAX study (14)], SGLT2 inhibitors [CANVAS study (15), DECLARE-TIMI 58 trial (16), EMPEROR-Preserved study (17)], and angiotensin receptor neprilysin inhibitors (ARNI) [PARAGON-HF trial (18)].

Most of these trials did not yield satisfactory results; however, the EMPEROR-Preserved study (17) is the first successful clinical trial for HFpEF. The study found that Empagliflozin significantly reduced the incidence of the composite endpoint of cardiovascular death or heart failure hospitalization (HHF) compared to placebo. The PARAGON-HF trial (18) did not show a statistically significant difference in primary endpoint events (cardiovascular death and heart failure hospitalization) compared to valsartan (*P* = 0.0585). Additionally, a trend of benefit was observed in two subgroups: women (HR = 0.73, 95% CI: 0.59–0.90) and LVEF ≤ 57% (HR = 0.78, 95% CI: 0.64–0.95). The 2023 Focused Update of the 2021 ESC Heart Failure Guidelines classified SGLT2 inhibitors as a class 1A recommendation (19). Enhancing the precision of treatment strategies through the development of specific drugs for HFpEF remains a crucial area of ongoing research.

HFpEF is a significant public health concern, and relying solely on modern medical treatments is insufficient for effective management. As the understanding of HFpEF in traditional Chinese medicine (TCM) grows, numerous scholars have conducted extensive studies on TCM treatment protocols and clinical efficacy evaluations for HFpEF. Their findings indicate that TCM offers distinctive advantages in improving clinical symptoms, cardiac function, and quality of life in HFpEF patients (20). A 2018 meta-analysis of 17 randomized controlled trials (RCTs) reported that TCM combined with western medicine effectively improved exercise tolerance and quality of life in HFpEF patients (21). The Chinese medicine QishenYiqi pill has been shown to improve cardiac function and inhibit myocardial fibrosis in HFpEF mice by reducing microvascular endothelial inflammation and activating the NO-cGMP-PKG pathway (22). Therefore, TCM's advantages can be fully utilized to intervene at various stages of HFpEF onset and progression, addressing multiple targets and pathways.

Although numerous clinical trials have been conducted to assess the efficacy and safety of TCM since the first RCT was published in 1982 (23), most current studies are small-scale, low-quality trials, case reports, or clinical experience reports. Additionally, the reliability of study results, report completeness, and their relevance for guiding clinical practice have been questioned (24). The evidence supporting TCM diagnosis and treatment remains widely unaccepted, limiting its broader promotion and application.

According to the Guidelines for Diagnosis and Treatment of Chronic Heart Failure in Traditional Chinese Medicine (2022) (25), the TCM syndrome types for HFpEF include Qi deficiency with blood stasis, Qi-Yin deficiency with blood stasis, and Yang-Qi deficiency with blood stasis. Through long-term treatment and observation of HFpEF patients, we found that they exhibit deficiencies in Qi, blood, Yin, and Yang, along with blood stasis, characterized by symptoms like palpitations, shortness of breath, weakness, exertional sweating, spontaneous or nocturnal sweating, cyanosis of the lips and tongue, and a thin, astringent, intermittent, or weak pulse. Therefore, Qi-Yin deficiency with blood stasis is the most common pattern, prompting us to develop the Fuzheng Yangxin Granule (FZYX). We plan to conduct a rigorously designed RCT to evaluate the efficacy and safety of FZYX in treating HFpEF with Qi-Yin deficiency and blood stasis syndrome.

## 2 Methods and analysis

### 2.1 Study design

**Figure 1:**
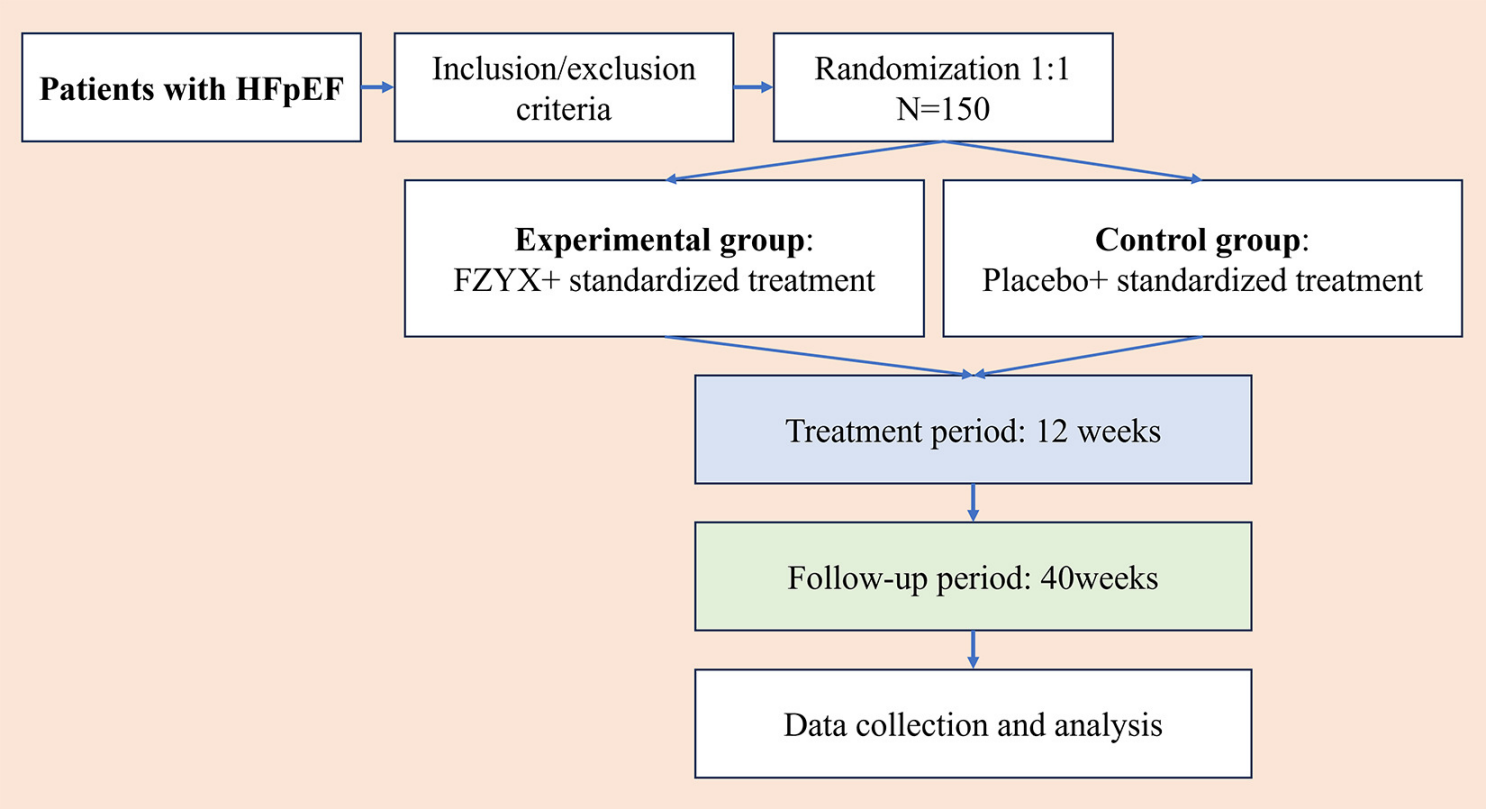
Flow chart of the trial. HFpEF, heart failure with preserved ejection fraction; FZYX, Fuzheng Yangxin Granule.

This study is designed as a randomized, double-blind, placebo-controlled, parallel-group, multicenter trial. The study design follows the Standard Protocol Items: Recommendations for Interventional Trials (SPIRIT) Guidelines (26). Seven tertiary hospitals across different regions of China are participating in this trial. A total of 150 stable HFpEF patients meeting the inclusion and exclusion criteria will be randomized 1:1 into either the experimental or control group. The overall design of the trial is presented in Figure 1.

### 2.2 Sample size estimation

The primary efficacy index of this trial is peak oxygen uptake (peak VO2), as demonstrated in previous related studies (27–29). It is anticipated that the peak oxygen uptake after treatment will be 16 ml/kg/min in the control group and 17.6 ml/kg/min in the experimental group, with a standard deviation of 3.1 ml/kg/min between the two groups. The sample size was calculated using PASS 15.0. The sample size was estimated by comparing the means of two samples in a completely randomized design, using a two-sided test with a significance level of *α* = 0.05 and power (1-*β*) of 0.80. With a 1:1 allocation between the experimental and control groups, the sample size for each group was estimated to be 60 participants. Considering a potential 20% attrition rate, 75 participants were included in each group, resulting in a total of 150 participants.

### 2.3 Inclusion criteria

1. Participants aged 18–80 years, irrespective of gender.
2. Participants diagnosed with Qi-Yin deficiency and blood stasis syndrome, as defined by Traditional Chinese Medicine (TCM) principles: Patients must present with at least two of three cardinal symptoms (shortness of breath/dyspnea, fatigue, or palpitations) combined with two of four secondary symptoms (persistent thirst/dry pharynx, spontaneous daytime sweating exacerbated by activity or night sweating ceasing upon awakening, heat sensation in palms/soles, or cyanotic facial/lip discoloration). Characteristic tongue manifestations include dark red or purplish coloration with ecchymosis/petechiae/varicose sublingual veins, thin body with scanty, absent, peeled, or fissured coating. Pulse findings must demonstrate thready-rapid-weak characteristics or irregular-intermittent rhythm. Syndrome confirmation requires concurrent fulfillment of symptom criteria and corresponding tongue-pulse presentations.
3. New York Heart Association (NYHA) functional class II-III with hemodynamic stability.
4. Meeting the diagnostic criteria for HFpEF as outlined in the National Heart Failure Guideline 2023 (30).
  (a) Epidemiological and population characteristics of HFpEF patients;
  (b) Signs and/or symptoms of heart failure;
  (c) LVEF ≥ 50%;
  (d) Objective evidence of cardiac structural and/or functional abnormalities consistent with left ventricular diastolic dysfunction and/or elevated left ventricular filling pressures. This includes: (i) E/e’ >15, (ii) Septal e’ <7 cm/s or Lateral e’ <10 cm/s, (iii) tricuspid regurgitant velocity >2.8 m/s or estimated PASP > 35 mmHg, (iv) elevated LAVI (sinus rhythm: LAVI >34 ml/m^2^, atrial fibrillation: LAVI > 40 ml/m^2^), and (v) elevated natriuretic peptide levels.
5. Voluntary participation, with participants providing informed consent after understanding the study details.

### 2.4 Exclusion criteria

1. Any prior echocardiographic LVEF measurement <40%.
2. Uncontrolled hypertension, defined as resting systolic blood pressure ≥180 mmHg and/or diastolic blood pressure ≥110 mmHg, confirmed at two separate examinations before randomization.
3. ALT, AST, or bilirubin levels exceeding 3 times the upper limit of normal (not due to heart failure), glomerular filtration rate <15 ml/min/1.73 m^2^, and blood potassium >5.5 mmol/L.
4. Within 3 months of: acute coronary syndrome, stroke, transient ischemic attack, cardiac, carotid, or other major vascular surgery, PCI, carotid angioplasty, coronary artery bypass grafting, or any non-cardiac condition that may impair exercise capacity or be exacerbated by strenuous exercise (e.g., infection, liver/kidney failure, thyrotoxicosis).
5. Patients with severe primary diseases affecting the liver, kidneys, hematopoietic, nervous, or endocrine systems, tumors, or psychiatric disorders.
6. Life expectancy of less than one year.
7. Known allergies to any trial medications.
8. Participation in other drug trials within the last month.
9. Patients currently using Chinese medicine or patent medicines containing ingredients similar to FZYX.
10. Pregnancy (confirmed by a positive test if needed), lactation, or women of childbearing age not using effective contraception.
11. Patients deemed unable to complete or comply with the study requirements, per investigator's judgement.

### 2.5 Randomization and blinding

A computer-generated blocked randomization scheme will be implemented using permuted blocks of 6 participants (3 FZYX:3 placebo) to ensure balanced allocation throughout recruitment. An independent statistician will generate the randomization sequence with consecutive numbering from 001 to 150 assigned to pre-packaged, identical medication bags. Participants will be sequentially enrolled and allocated medications strictly according to their entry order, with each bag containing either FZYX or placebo labeled only with the corresponding randomization code. The allocation list will remain securely encrypted in a password-protected file accessible solely to the independent statistician until database lock. This double-blind design ensures concealment from participants, investigators, outcome assessors, and data analysts throughout the trial duration.

### 2.6 Intervention

Following randomization, participants will receive either FZYX or matched placebo orally (2 sachets three times daily) for 12 weeks, with both preparations manufactured under Good Manufacturing Practice (GMP) standards at Xiyuan Hospital's pharmaceutical facility. The investigational products will be distributed through a serialized tracking system managed by the principal investigator, with documentation of batch numbers, dispensing dates, and return quantities. All participants will concurrently receive guideline-directed standardized medical therapy including: (1) sodium-glucose cotransporter-2 inhibitors (SGLT2i: empagliflozin/dapagliflozin) for HFpEF management; (2) diuretics for fluid-overloaded patients (NYHA II-IV); (3) angiotensin receptor-neprilysin inhibitors (ARNI: sacubitril/valsartan) indicated for symptomatic females [any left ventricular ejection fraction (LVEF)] or males (LVEF <55%–60%); (4) mineralocorticoid receptor antagonists (MRA: spironolactone) indicated for symptomatic females (any LVEF) or males (LVEF <55%–60%); (5) stable regimens for comorbidities (e.g., hypertension/diabetes). FZYX's herbal composition (see Table 1) and placebo share identical organoleptic properties through standardized production protocols.

**Table 1:** Composition of FZYX.

Herbal name	Latin name	Dosage (g)
Ren Shen	Ginseng Radix Et Rhizoma	20
Huang Qi	Astragali Radix	30
Mai Dong	Ophiopogonis Radix	20
Di Huang	Rehmanniae Radix	15
Dang Gui	Angelicae Sinensis Radix	15
Hong Jing Tian	Rhodiolae Crenulatae Radix Et Rhizoma	30
Fu Zi	Aconiti Lateralis Radix Praeparata	10
San Qi	Notoginseng Radix Et Rhizoma	6

### 2.7 Study visits and follow-up

The investigator will obtain informed consent from each participant before initiating any protocol-related procedures. Eligible participants will be contacted by telephone and scheduled to attend the trial center within one week (Visit 1). During Visit 1 and 4, the investigator will collect relevant information, including demographic data, medical history, medication use, and conduct a physical examination. Participants will then be randomized into either the experimental or control group. In this trial, participants will take FZYX or placebo three times a day for 12 weeks. All participants will undergo scheduled testing and evaluations, including cardiopulmonary exercise testing (CPET), echocardiography, 12-lead electrocardiogram (ECG), routine blood tests, routine urine tests, and blood biochemical tests, including N-terminal pro-B-type natriuretic peptide (NT-proBNP), serum electrolytes, liver and kidney function assessments, coagulation indices, and hemorheology examination. In Visits 2 and 3, participants will undergo the same tests and evaluations as in Visit 1, except for echocardiography and CPET. Follow-up visits 1–8 will consist of telephone follow-ups to record Minnesota Living with Heart Failure Questionnaire (MLHFQ) scores, FRAIL scale scores, all-cause mortality, and composite endpoint events. The detailed follow-up schedule is shown in Table 2.

**Table 2:** Measurement items and points of data capture.

Items	Lead-in period	Treatment period				Follow-up period	
	-7∼-1D	-1∼0D	4W ± 3D	8W ± 3D	12W ± 3D	16,20,24,28W ± 5D	34,40,46,52W ± 5D
	Screening	Visit 1	Visit 2	Visit 3	Visit 4	Follow-up 1–4	Follow-up 5–8
Patients							
Eligibility screen	✓						
Informed consent	✓						
Relevant information^a^	✓						
Allocation	✓						
Outcomes							
CPET		✓			✓		
Echocardiography		✓			✓		
ECG		✓	✓	✓	✓		
Routine blood test		✓	✓	✓	✓		
Blood biochemical test^b^		✓	✓	✓	✓		
Routine urine test		✓	✓	✓	✓		
NYHA functional class		✓	✓	✓	✓		
6-MWT		✓	✓	✓	✓		
TCM syndrome score		✓	✓	✓	✓		
MLHFQ score		✓	✓	✓	✓	✓	✓
FRAIL scale		✓	✓	✓	✓	✓	✓
Composite endpoint event		Record at any time					
All-cause mortality							
Adverse event							

a Related Information includes: demographic data, medical history, medication use.

b Blood biochemical tests, includes: N-terminal pro-B-type natriuretic peptide (NT-proBNP), serum electrolytes, liver and kidney function assessments, coagulation indices, and hemorheology examination; D, day; W, week.

### 2.8 Outcome measures

#### 2.8.1 Primary outcome

The primary evaluation measure is peak VO_2_.

#### 2.8.2 Secondary outcomes

(1) All-cause mortality;
(2) Composite endpoint events (treatment abandoned due to worsening heart failure, successful resuscitation after cardiac arrest, malignant arrhythmia, nonfatal stroke, heart failure hospitalization);
(3) 6-minute walking distance (6MWD);
(4) NYHA functional class;
(5) Serum NT-proBNP levels;
(6) Echocardiography: LVEF, E/e', and others;
(7) TCM syndrome scores;
(8) Minnesota Living with Heart Failure Questionnaire (MLHFQ) scores;
(9) Blood rheology (fibrinogen, whole blood viscosity, platelet aggregation test, erythrocyte sedimentation rate, and others);
(10) FRAIL scale.

### 2.9 Safety assessment and adverse events report

Detailed observation and reporting of all types of drug-related allergic reactions (including rash), gastrointestinal reactions (such as nausea, vomiting, diarrhea), neuropsychiatric disorders (including insomnia, headache, dizziness), and abnormal laboratory test results. Laboratory tests include routine blood tests (white blood cell count, red blood cell count, hemoglobin, platelets), routine urine tests (urine leukocytes, urine protein, occult blood, urine glucose), liver function tests (alanine transaminase, aspartate transaminase, total bilirubin, gamma-glutamyl transferase, alkaline phosphatase), renal function tests (serum creatinine, blood urea nitrogen), serum electrolytes (serum potassium, sodium, chloride), and 12-lead ECG. Any serious adverse events occurring during the trial must be immediately reported to the principal investigator and the ethics committee. Additionally, a “Serious Adverse Event” form must be completed.

### 2.10 Statistical collection and analysis

Study data will be collected at each center using standardized paper-based Case Report Forms (CRFs). Participants will be provided with a private space to complete the quality of life form to address privacy concerns. Two-tailed tests will be applied with *P* < 0.05 as the significance threshold. No adjustments for multiple testing will be performed for secondary endpoints, as they are exploratory. Analyses will be conducted using SPSS 23.0 and R 4.3.1. Primary outcome: Analyzed using ANCOVA adjusted for baseline peak VO₂, age, and sex. Secondary continuous outcomes: Assessed via mixed-effects models for repeated measures. Non-normally distributed data will use bootstrap CIs (10,000 replications). Binary/categorical data: Analyzed using chi-squared or Fisher's exact test. Ordinal outcomes: Evaluated via proportional odds models.

### 2.11 Study monitoring and quality assurance

The study will adhere to the clinical trial protocol, the Declaration of Helsinki, and applicable Chinese clinical research regulations. Investigators will design CRFs to document data related to trial participants. All investigators will receive standardized training to ensure a comprehensive understanding of the clinical trial before its initiation. Investigators will ensure that all reported trial data are accurate, complete, and verifiable against source documents. A dedicated monitor will supervise the entire trial process, regularly verifying that the trial is conducted and documented according to the protocol, standard operating procedures, and applicable regulations.

## 3 Discussion

HFpEF, a highly heterogeneous clinical syndrome, has become a significant type of heart failure globally. The pathophysiology of HFpEF is associated with several factors, including chronic systemic inflammation, microvascular dysfunction, metabolic disorders, myocardial fibrosis, epicardial adipose tissue accumulation, and neuroendocrine system activation (31). Moreover, non-cardiovascular comorbidities, such as chronic kidney disease, diabetes mellitus, and obesity, are more prevalent in this population (32). The prevalence of HFpEF is expected to rise in the coming years, driven by population aging and the increasing prevalence of comorbidities associated with the condition. The complex pathophysiology and heterogeneity of clinical phenotypes in HFpEF are key factors contributing to the failure of many clinical trials, making it a challenging condition to treat.

The clinical need for HFpEF is significant and unmet, with TCM research showing promise. Given its unique framework and extensive experience, TCM warrants further exploration for HFpEF treatment. Qi-Yin deficiency with blood stasis is the most common TCM syndrome in HFpEF. FZYX, suited for this syndrome, consists of Ren Shen, Huang Qi, Mai Dong, Di Huang, Dang Gui, Hong Jing Tian, Fu Zi, and San Qi. In this formula, Ren Shen and Huang Qi benefit Qi; Mai Dong, Di Huang, and Dang Gui nourish Yin and promote fluids; Fu Zi (in small amounts) warms Yang and benefits Qi; Dang Gui and San Qi invigorate blood and remove stasis.

In our clinical practice (33–36), we studied TCM prescriptions like FZYX combined with hemofiltration in heart failure patients with diuretic resistance. Studies showed that patients receiving hemofiltration with TCM had lower rates of hypokalemia, hyponatremia, and hypotension compared to those without TCM. Additionally, heart function improved more significantly in patients treated with TCM. These positive results in treating severe heart failure suggest promising applications for TCM in broader heart failure treatment. In a previous experiment, we found that FZYX improves cardiac function and protects cardiomyocytes by regulating STAT3 expression and inhibiting apoptosis in a heart failure rat model (37).

Previous studies of TCM for HFpEF have encountered three major issues. First, many face methodological flaws. The current RCT designs for TCM in HFpEF lack rigor, with improper execution of randomization, blinding, allocation concealment, and reporting biases, all of which undermine study credibility. Secondly, some trials rely solely on modern diagnoses, neglecting TCM symptom selection, which hinders subject “homogenization” and impacts clinical efficacy. Modern medicine classifies by disease, while TCM uses symptom differentiation. Integrating both is essential for standardizing research subjects. Third, hard endpoints like cardiovascular death and rehospitalization are often overlooked (38). Prognostic indicators require long observation periods and significant resources. In contrast, easier-to-detect, shorter-term indicators are commonly used (39). Yet, given HFpEF's severity, more trials should focus on mortality as the primary endpoint.

This study has three key strengths justifying the trial. First, the protocol follows SPIRIT guidelines, ensuring high-quality clinical evidence. Second, patient diagnoses combine modern HFpEF criteria with TCM's Qi-Yin deficiency and blood stasis syndrome, integrating disease and syndrome. Third, the study evaluates long-term prognostic outcomes in addition to objective HFpEF tests. In conclusion, this multicenter, randomized controlled trial evaluates FZYX for HFpEF treatment, aiming to provide high-quality, evidence-based support for its efficacy and safety. Additionally, this study integrates Chinese and Western medicine, potentially guiding alternative treatment strategies.

## Ethics statement

The Ethics Committee of Xiyuan Hospital, China Academy of Chinese Medicine, approved this trial protocol on May 21, 2024 (2024XLA083-1). Any deviations from the protocol require prior approval from the Ethics Committee. The trial is registered in the Chinese Clinical Trials Registry (ChiCTR2400087293). The investigator must provide all subjects with a detailed explanation of the study and obtain informed consent. Subjects will be given sufficient time to make an informed decision regarding their participation in the trial.
