# Correction: A multi-center, randomized, double-blind, placebo-controlled trial to evaluate the efficacy and safety of Fuzheng Yangxin Granule in treating heart failure with preserved ejection fraction (Qi-Yin deficiency and blood stasis syndrome): study protocol

**DOI:** 10.3389/fcvm.2026.1854465

**Published:** 2026-05-06

**Authors:** Jingjing Chen, Zian Yan, Jiacong Wang, Lijun Guo, Zhonghui Jiang, Fangfang Wang, Ruina Bai, Xiaochang Ma

**Affiliations:** 1 School of Clinical Medicine, Xiyuan Hospital, Beijing University of Chinese Medicine, Beijing, China; 2 Cardiovascular Department, Xiyuan Hospital, China Academy of Chinese Medical Sciences, Beijing, China; 3 Cardiovascular Disease Center, Xiyuan Hospital, National Clinical Research Center for Chinese Medicine Cardiology, Beijing, China

**Keywords:** heart failure with preserved ejection fraction, Fuzheng Yangxin Granule, traditional Chinese medicine, clinical trial protocol, herbal medicine, randomized controlled trial

Affiliation 1 School of Clinical Medicine, Xiyuan Hospital, Beijing University of Chinese Medicine, Beijing, China; 2 Cardiovascular Department, Xiyuan Hospital, China Academy of Chinese Medical Sciences, Beijing, China; 3 Cardiovascular Disease Center, Xiyuan Hospital, National Clinical Research Center for Chinese Medicine Cardiology, Beijing, China were erroneously given as 1 Cardiovascular Department, Xiyuan Hospital, China Academy of Chinese Medical Sciences, Beijing, China; 2 Cardiovascular Disease Center, Xiyuan Hospital, National Clinical Research Center for Chinese Medicine Cardiology, Beijing, China; 3 Graduate School, Beijing University of Chinese Medicine, Beijing, China.

The original version of this article has been updated.

